# An Improved Electromagnetic Field Optimization for the Global Optimization Problems

**DOI:** 10.1155/2019/6759106

**Published:** 2019-05-23

**Authors:** Alkin Yurtkuran

**Affiliations:** Dept. of Industrial Engineering, Bursa Uludag University, Bursa, Turkey

## Abstract

Electromagnetic field optimization (EFO) is a relatively new physics-inspired population-based metaheuristic algorithm, which simulates the behavior of electromagnets with different polarities and takes advantage of a nature-inspired ratio, known as the golden ratio. In EFO, the population consists of electromagnetic particles made of electromagnets corresponding to variables of an optimization problem and is divided into three fields: positive, negative, and neutral. In each iteration, a new electromagnetic particle is generated based on the attraction-repulsion forces among these electromagnetic fields, where the repulsion force helps particle to avoid the local optimal point, and the attraction force leads to find global optimal. This paper introduces an improved version of the EFO called improved electromagnetic field optimization (iEFO). Distinct from the EFO, the iEFO has two novel modifications: new solution generation function for the electromagnets and adaptive control of algorithmic parameters. In addition to these major improvements, the boundary control and randomization procedures for the newly generated electromagnets are modified. In the computational studies, the performance of the proposed iEFO is tested against original EFO, existing physics-inspired algorithms, and state-of-the-art meta-heuristic algorithms as artificial bee colony algorithm, particle swarm optimization, and differential evolution. Obtained results are verified with statistical testing, and results reveal that proposed iEFO outperforms the EFO and other considered competitor algorithms by providing better results.

## 1. Introduction

Nowadays, the use of metaheuristic algorithms has surprisingly increased for solving various problems due to their flexibility, gradient-free mechanism, and local optima avoidance structures [1]. Although they do not guarantee the optimal solution for an optimization problem, they mostly have a capability of finding the near-optimal solution. During the last few decades, various algorithms have been proposed to solve different real-life problems. In spite of the fact that most of them show similar behavior while searching the solution space, each algorithm may have superior performance on a specific problem as described in “No Free Lunch” theorem, which states that no single algorithm can perform well on every optimization problem [2].

**Figure 1:**
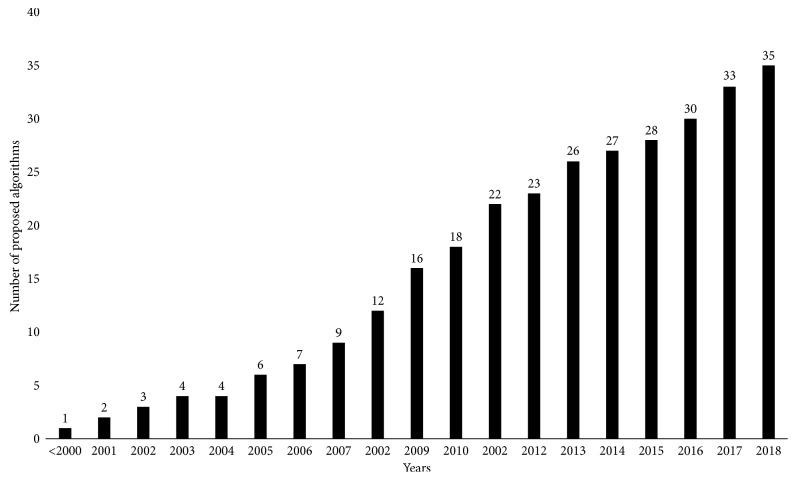
Cumulative numbers of the proposed physics-inspired metaheuristic algorithms by the years.

Nature-inspired optimization algorithms have been widely adopted in the area of computer science, mathematics, control, or decision making due to their efficient performance on solving complex optimization problems since the last few decades [3]. Considering the existing literature, different classifications for the nature-inspired metaheuristic algorithms are given based on a specific philosophy. These algorithms are simply classified into three main groups: evolution-inspired, physics-inspired, and swarm-inspired [4–6]. Among this classification, physics-inspired algorithms simulate physical laws in the universe, and they are different from other approaches because of their search agents based on physics rules [7, 8]. A considerable number of metaheuristic algorithms in the literature have taken inspiration from physical phenomena. A detailed review of the physics-inspired metaheuristic algorithms are given in the study of Can and Alatas [9] and Tahani and Babayan [10], where the list of the algorithms reviewed in these studies is presented in Table 1. Moreover, Table 1 also includes the algorithms, which are not given in both the studies. Considering the list of the physics-inspired metaheuristic algorithms, cumulative numbers of the algorithms by the years are given in Figure 1. It should be noted from Figure 1 that most of these algorithms have been proposed in the last decade.

Regarding the physics-inspired metaheuristic algorithms given in Table 1, the electromagnetic field optimization (EFO) is one of the relatively new physics-inspired metaheuristic algorithms, which is first proposed by Abedinpourshotorban et al. [14]. The EFO is inspired by the behavior of electromagnets with different polarities and takes advantage of a nature-inspired ratio, known as the golden ratio. In EFO, the population consists of electromagnetic particles made of electromagnets corresponding to variables of the optimization problem and is divided into three fields: positive, negative, and neutral. In each iteration, a new electromagnetic particle is generated based on the attraction-repulsion forces among these electromagnetic fields, where the repulsion force helps the particle to avoid the local optimal point and the attraction force leads to find global optimal point.

Since the EFO is a recently proposed algorithm, the number of studies that consider this approach is limited. Yurtkuran and Kucukoglu [44] used the original version of the EFO and also other three different physics-inspired metaheuristic algorithms such as electromagnetism-like algorithm, gravitational search algorithm, and weighted superposition attraction algorithm for the solar cell parameter estimation problem to optimize the performance of solar systems. The authors compared the performance of the algorithms on a well-known benchmark problem set. Their computational studies show that EFO outperforms the other three algorithms and provides better results. Bouchekara et al. [45] proposed a modified version of the EFO to identify optimal coordination of directional overcurrent relays for power systems protection, which is a nonlinear and highly constrained optimization problem. The authors applied two simple modifications on the algorithm by changing the uniformly distributed random generation procedure (used in the search equation) to the normal distributed random generation and boundary check procedure of the electromagnets. In computational studies, the efficiency of the modified version of the EFO is shown. Talebi and Dehkordi [46] introduced a binary version of the EFO for the sensitive association rules hiding for personal information protection. Performance of the algorithm is evaluated by doing experiments on both real-world and synthetic datasets. Better results are observed by binary EFO comparing with four different algorithms.

In general, metaheuristic algorithms are established to balance two important factors for their performance, which are exploration and exploitation. The exploration means the ability to investigate in the unvisited regions of the search space, whereas exploitation refers to the ability to search around better solutions [47]. However, the lack of the right balance between these factors leads to poor convergence when solving complex problems. Therefore, many studies have been made to improve the performance of metaheuristic algorithms based on three strategies [48]: (i) hybridization of the algorithms [49–51], (ii) adaptation mechanism [52–54], and (iii) introducing new operators for generating new solutions [55–57]. In this manner, this paper introduces an improved version of the EFO, which is called improved electromagnetic field optimization (iEFO). Concerning the original EFO, the iEFO has two novel changes: new solution generation function for the particles and adaptive control of algorithmic parameters. With the new solution procedure, the selection probability of the better solutions in the candidate particle generation step is increased. Furthermore, by an adaptive control mechanism, it explores the search space effectively, especially in the early stages of the search process, whereas exploitation is emphasized in the latter phases. In addition to these major improvements, the boundary control and randomization procedures for the newly generated particles are modified.

A modified version of the EFO is introduced by Bouchekara et al. [45]. However, the modified version of the EFO proposed by the authors includes minor changes in random number generation and boundary condition in the new electromagnet generating step. To the best of our knowledge, this paper is the first improved version of the EFO with major changes on the search mechanism, new solution generation, and control of search parameters. The main contributions of the proposed study can be summarized as follows: (i) a new search equation is defined to balance the exploration and exploitation performance, (ii) in order to further improve the exploitation behavior, the parameters of the new search equation are controlled adaptively, and (iii) a detailed literature review of physics-inspired metaheuristic algorithms is presented. In computational studies, the performance of the proposed iEFO is analyzed on a well-known global optimization benchmark problems into three cases. First, the proposed iEFO is compared with the original version of the EFO and also four recent physics-inspired population-based metaheuristic algorithms. Second, the proposed iEFO is compared with original version of the well-known artificial bee colony (ABC) algorithm [58], differential evolution (DE) algorithm [59], and particle swarm optimization (PSO) algorithm [60]. Third, the proposed iEFO is compared with the improved variants of PSO and DE, and detailed statistical analyses for the performance comparisons are given in the computational studies.

The remainder of the paper is organized as follows. In Section 2, the original version of the EFO and its main steps are described.  Section 3 introduces the proposed iEFO for global optimization problems. Computational results are given in Section 4. Finally, a conclusion part of future research perspectives is provided in Section 5.

## 2. Electromagnetic Field Optimization (EFO)

The EFO is a relatively new metaheuristic algorithm inspired by attraction-repulsion forces among electromagnets with different polarities and nature-inspired ratio called the golden ratio [14]. In EFO, a solution is represented by electromagnetic particle (EMP) made of electromagnets, and the number of electromagnets is equal to the number of variables in the optimization problem. Different from permanent magnets, each electromagnet in EMP has the same single polarity (positive or negative), and each electromagnet can apply a force of attraction or repulsion among other neighbor electromagnets.  Table 2 summarizes the notations used in the EFO. In this context, the main steps of the algorithm are described in this section.

### 2.1. Initialization

As in most of the population-based metaheuristic algorithms, the EFO starts by randomly generating a population of electromagnetic particles by using equation (1). Each electromagnet of an EMP is randomly generated within its lower and upper bounds. After a population randomly generated, EMPs are sorted based on their fitness value in descending order:

(1) $$\begin{array}{c} {\text{EMP}}_{j}^{i}=L_{j}+\text{rand}\left(U_{j}-L_{j}\right),\;\;i=1,\ldots ,N\_\text{emp},j=1,\ldots ,N\_\text{var.} \end{array}$$

### 2.2. Classification

In the classification phase of the EFO, the EMPs are classified into three groups with different polarities: positive field that contains the best EMPs, negative field that contains worst EMPs, and neutral field that contains small negative polarities. The number of EMPs of these three groups is determined by using two control parameters: *P*_field and *N*_field. The *P*_field and *N*_field represent the percentage of the allocated solution for positive and negative part, respectively. The remaining solutions form the neutral part. According to the classification of the population, selection of one EMP from each field for the new electromagnet generation procedure is made by using the following equations:

(2) $$\begin{array}{c} P_{j}=\text{rnd}\_\text{int}\left(1,\left\lfloor N\_\text{emp}*P\_\text{field}\right\rfloor \right),\;j=1,\ldots ,N\_\text{var,}\\ N_{j}=\text{rnd}\_\text{int}\left(\left\lfloor N\_\text{emp}*\left(1-N\_\text{field}\right),N\_\text{emp}\right\rfloor \right),\;\;j=1,\ldots ,N\_\text{var,}\\ K_{j}=\text{rnd}\_\text{int}\left(\left\lceil N\_\text{emp}*P\_\text{field}\right\rceil ,\left\lceil N\_\text{emp}*\left(1-N\_\text{field}\right)\right\rceil \right),\;\;j=1,\ldots ,N\_\text{var}. \end{array}$$

### 2.3. New Solution Generation

New solution generation procedure is the most important step of the EFO. After specifying the randomly selected indexes from positive, neutral, and negative fields, a new electromagnet for the candidate solution is generated by using equation (5) as follows. If a uniformly distributed random number is lower than the *P*s_rate, then the corresponding electromagnet of the candidate solution is set to the randomly selected electromagnet from the positive field. Otherwise, the corresponding electromagnet of the candidate solution is generated based on the randomly selected electromagnet from the neutral field, which is affected by the randomly selected electromagnets from positive and negative fields:

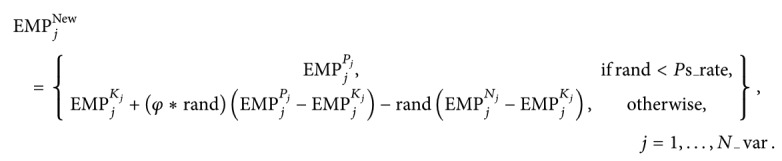
(3)


After an electromagnet is generated for the new solution, its bounds are checked whether it is in the range of lower and upper limit. If the new electromagnet violates its bounds, then it is regenerated randomly within its bounds as in the initialization step.

### 2.4. Randomization

In each iteration, the randomization step is applied to change only one electromagnet of generated EMP with randomly generated electromagnet within its range as follows:

(4) $$\begin{array}{c} {\text{EMP}}_{\text{RI}}^{\text{New}}=L_{\text{RI}}+\text{rand}\left(U_{\text{RI}}-L_{\text{RI}}\right),\;\text{if rand}<R\_\text{rate,} \end{array}$$

where RI is an integer counter used to identify the index of the electromagnet for randomization. RI is initialized with 1 or randomly generated integer number between [1, *N*_var] at the beginning and is increased by 1 in each randomization process. In case the RI reaches to its upper limit *N*_var, then it is set to 1 again.

### 2.5. Selection

At the end of each iteration, the fitness function value of the new solution is determined. If the generated EMP provides a better solution than the worst EMP (*N*_emp^th^ particle) in the population, then the new solution is inserted into the sorted population according to its fitness function value and the worst EMP is removed from the population.

## 3. Proposed iEFO

Generally, metaheuristic algorithms try to balance the two important factors for their performance as exploration and exploitation. The candidate solution generation mechanism (search equation) plays an important role in determining the performance of a metaheuristic algorithm [61]. Up to now, researches have focused on different search equation modifications to improve the performance of well-known algorithms such as ABC, PSO, and DE. Inspired by these studies, a novel search equation is defined for iEFO in this study. As can be seen in equation (5), the original EFO uses the EMP from the neutral field and generate a new candidate solution using this EMP with positive feedback from the positive field, whereas negative feedback from the negative field. In the iEFO, a new search equation is proposed as follows:

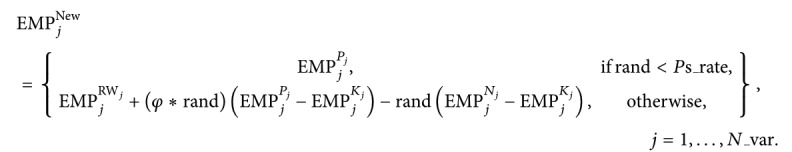
(5)


Similar to the original EFO, *P*_*j*_, *N*_*j*_, and *K*_*j*_ are the indexes of the selected EMPs from positive, negative, and neutral parts, respectively. *φ* is the golden ratio constant, which is used to guide the candidate solutions towards the positive part. Here, RW_*j*_ depicts the selected EMP using the roulette wheel method, which is different from *P*_*j*_, *N*_*j*_, and *K*_*j*_. In other words, in the iEFO, the candidate EMP is constructed based on the EMP, which is selected from the roulette wheel. Equation (7) can generate the candidate solution not only around the neutral field but also positive and negative fields, while better EMPs have higher probability to be selected. It is clear that guiding the search by RW_*j*_ will improve the exploitation ability of EFO.

Next, to further improve the exploitation performance of iEFO, an adaptive mechanism is employed. It is well known that integrating adaptive control mechanisms into metaheuristic algorithms is a very popular technique in the related literature [52, 54, 62]. In iEFO, two main control parameters, i.e., *P*s_rate and *R*_rate, are controlled adaptively over the course of a run. As mentioned in the previous section, *P*s_rate is responsible for the probability of copying the index of the EMP from the positive field, whereas *R*_rate is the probability of the randomization procedure. In the iEFO, *P*s_rate and *R*_rate are updated at the end of each iteration as follows:

(6) $$\begin{array}{c} P\text{s}\_\text{rate}=P{\text{s}}_{\text{RMin}}+\frac{\text{Iter}\times \left(P{\text{s}}_{\text{RMax}}-P{\text{s}}_{\text{RMin}}\right)}{\text{MaxIter}}, \end{array}$$

(7) $$\begin{array}{c} R\_\text{rate}=R_{\text{RMax}}-\frac{\text{Iter}\times \left(R_{\text{RMax}}-R_{\text{RMin}}\right)}{\text{MaxIter}}, \end{array}$$

where Iter and MaxIter refer to the current iteration value and the maximum iteration value, respectively. As can be seen from equation (6), *P*s_rate is increased from *P*s_RMin _ to *P*s_RMax_ during the search process. Similarly, *R*_rate is reduced adaptively from *R*_RMax_ to *P*s_RMin_. Here, *P*s_RMin _, *P*s_RMax_, *R*_RMax_, and *R*_RMin_ are the new control parameters of iEFO, which will be set before the search process. In a word, the two new control equation of *P*s_rate and *R*_rate will improve the exploration-exploitation balance by giving a higher probability to random search mechanism in the early phase, while candidate EMPs are more likely to be derived from the positive field at the latter phases of the search as *P*s_rate increases. In other words, iEFO will efficiently explore the search space during the early stages and will favor exploitation around the better solutions in the latter phases.

Finally, the iEFO employs a modified boundary check and a randomization mechanism. In the modified boundary check procedure, the electromagnets that are generated outside the boundaries are set back to the boundary values. In addition to the modification on boundary check procedure, in the random search step of the iEFO, the randomly selected EMP is regenerated within limits instead of a sequence-based approach.

**Figure 2:**
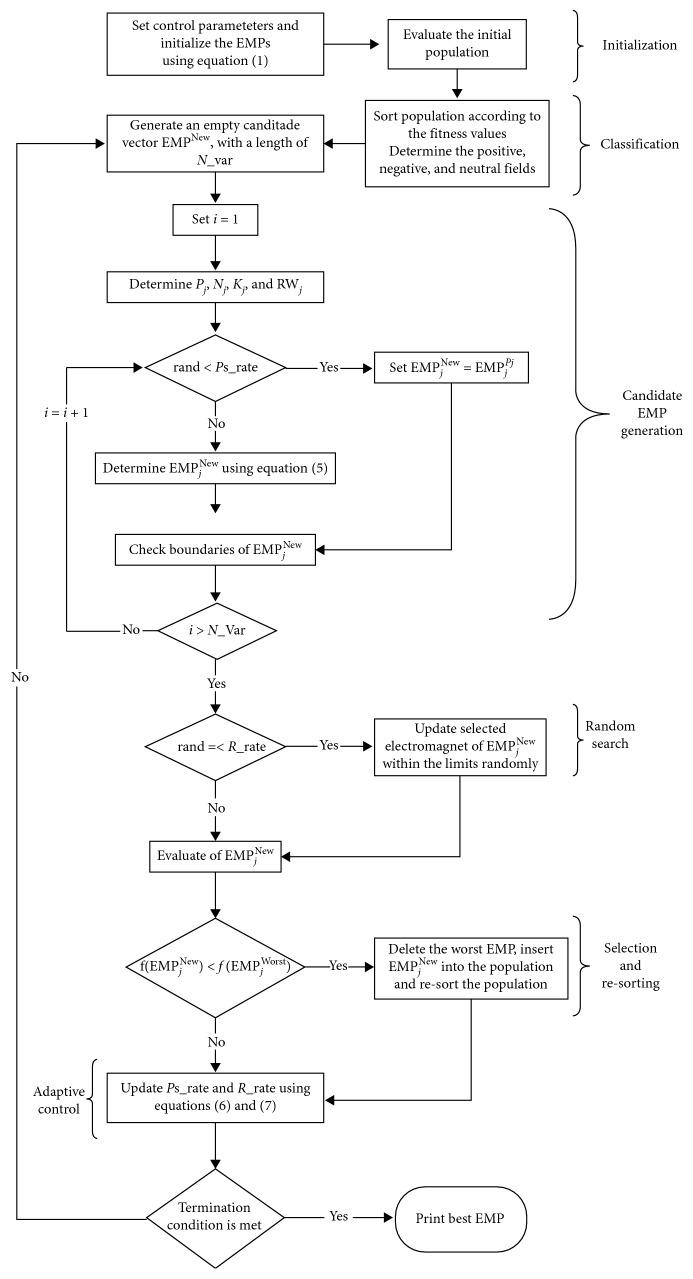
Flow chart of the proposed iEFO.

As a result of the descriptions given above, Figure 2 shows the flow chart of the iEFO and illustrates the main steps of the algorithms, i.e., initialization, classification, candidate EMP generation, randomization procedure or random search, selection, and re-sorting, and the adaptive control mechanism of *P*s_rate and *R*_rate.

**Figure 3:**
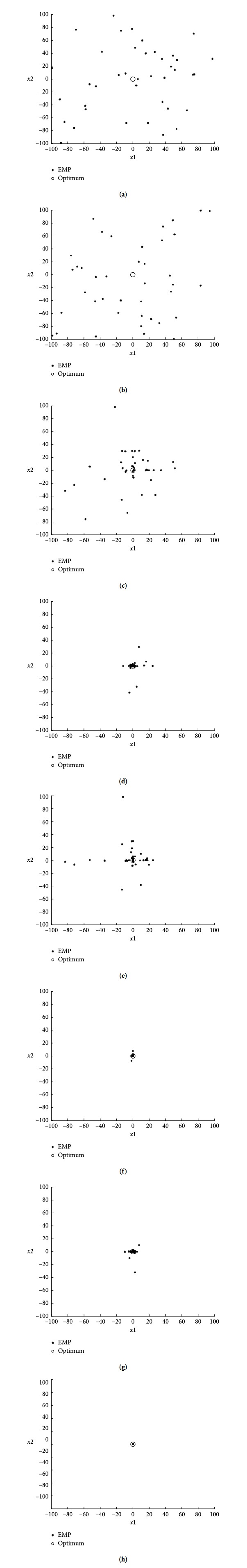
The convergence behavior of EFO and iEFO on Sphere function. (a) Population distribution at iteration = 1 for EFO. (b) Population distribution at iteration = 1 for iEFO. (c) Population distribution at iteration = 10 for EFO. (d) Population distribution at iteration = 10 for iEFO. (e) Population distribution at iteration = 20 for EFO. (f) Population distribution at iteration = 20 for iEFO. (g) Population distribution at iteration = 30 for EFO. (h) Population distribution at iteration = 30 for iEFO.

Before comparing iEFO against various algorithms, the distribution of EMP's during the search process at various stages of EFO and iEFO on the sphere function $\left(f\left(\vec{X}\right)=\sum_{i=1}^{D}x_{i}^{2}\right)$ is analyzed and shown in Figure 3. It should be noted that here, the parameter settings of EFO are set as described in [14], whereas preliminary parameter settings are used for iEFO. From Figure 3, it can be concluded that the convergence performance of iEFO is better than the EFO. Specifically, the guidance of RW_*j*_ and the adaptive mechanism of iEFO improve the convergence behavior of the population.

## 4. Computational Results

In this section, the performance of the iEFO is analyzed and tested against various well-known metaheuristic algorithms using three sets of experiments. First, iEFO is compared with the original EFO and other recent physics-inspired algorithms, such as gravitational search algorithm (GSA) [29], electromagnetism-like algorithm (EMA) [40], central force optimization algorithm (CFO) [34], and weighted superposition attraction algorithm (WSA) [13] on different benchmark problems with various dimensions. Second, iEFO is tested against ABC, PSO, and DE using the same problem set. In order to make fair comparisons, all algorithms used in the first two sets of experiments are coded and executed in the same environment. All algorithms conduct 30 independent runs on each benchmark function, and the maximum number of function evaluation (MaxFE) is employed as the termination criterion, which is set to 320,000 for all simulations. Further, all algorithms have been simulated in the MATLAB environment and executed on the same computer with Intel Xeon CPU (2.67 GHz) and 16 GB of memory. In the last part of the experiments, the performance of the iEFO is tested against powerful variants of PSO and DE. For this part, the competitor algorithms are fully informed particle swarm (FIPS) [63], self-organizing hierarchical PSO with time-varying acceleration coefficients (HPSO-TVAC) [64], comprehensive learning PSO (CLPSO) [57], self-adapting DE (JDE) [52], adaptive DE with optional external archive (JADE) [65], and self-adaptive DE (SaDE) [62]. In this subsection, the reported results from the corresponding studies are used for comparisons.

### 4.1. Benchmark Functions and Parameter Settings

In order to evaluate the performance of iEFO, 13 commonly used benchmark functions [1, 5, 6, 13, 47, 48, 61, 66–72] with various dimensions, which are listed in Table 3, are used. In Table 3, *D* refers to the dimension of the problem. To be specific, F1 and F8 are unimodal and nonseparable functions (UN), F2, F3, F5, F6, F10, and F13 are multimodal and separable functions (MS), F4, F9, and F12 are multimodal and nonseparable (MN), and F7 and F11 are unimodal and separable functions (US). Unimodal functions have one local minimum as the global optimum, whereas multimodal functions have more than one local optimum and the number of their local optimums increases with the problem dimension exponentially. On the other hand, separable functions can be written as sum of *n* functions with one variable, while nonseparable functions cannot be reformulated as the sum of subfunctions [47]. Further, F7–F10 are shifted functions and *o*, a shifted vector, is generated randomly in the corresponding search range. The name, formulation, type, search space (range), and the global optimum objective function values (*f*(*x* ^*∗*^)) are given in Table 3.

It is worth to mention that parameter settings play a vital role in the performance of a metaheuristic algorithm [73]. The parameters of the adaptive mechanism are set to the lower and upper limits given in [REF] as *P*s_RMin_=0.1, *P*s_RMax_=0.4, *R*_RMax_=0.4, and *R*_RMin_ = 0.1. Further, preliminary tests are carried out to determine the positive and negative field ratios within the range of *P*_Field_=[0.05, 0.10] and *N*_Field_=[0.45, 0.50]. According to the preliminary tests, *P*_Field_ and *N*_Field_ are set to 0.05 and 0.45, respectively. Additionally, the population size (*N*_emp) is set to 80.  Table 4 presents the control parameters of all competitor algorithms. In Table 4, PopSize refers to population size of each algorithm. It should be noted that the parameter settings of the competitor algorithms are set as the original papers or published codes.

### 4.2. Comparison with Physics-Inspired Algorithms

To demonstrate the effectiveness of iEFO, in this subsection, the iEFO is compared with physics-inspired algorithms as EFO, GSA, EMA, CFO, and WSA. Tables 5 [Table tab6]–7 present the experimental results on benchmark problems with *D*=  50, *D*=100, and *D*=200. In Tables 5 [Table tab6]–7, results are given in terms of mean and standard deviation (StdDev) achieved from 30 independent runs. For a precise and pairwise comparison, the statistical significance of the differences between iEFO and competing test algorithms are determined using *t*-tests with a significance level of 0.05. In Tables 5 [Table tab6]–7, “+” shows that iEFO is significantly better than the compared algorithm, “≈” indicates that there is no significant difference between the algorithms. Last, “−” depicts that the competitor algorithms achieve better results at a level of 0.05 significance. Additionally, the overall results are given in the last rows of Tables 5 [Table tab6]–7.

As can be seen from Table 5, on the problems with *D*=50, iEFO is better than the test algorithms on the majority of the benchmarks. To be more specific, iEFO performs significantly better than EFO, GSA, EMA, CFO, and WSA on 6, 8, 10, 9, and 9 out of 13 functions, respectively. On F7 and F11, all algorithms obtained statistically similar results. iEFO and EFO perform similar performance on F3, F4, F5, F7, F10, F11, and F13, whereas there is no significant difference between iEFO and GSA on F7, F8, F10, F11, and F12.

On the problems with *D*=100 (Table 6), only WSA can outperform iEFO on F2 with *D*=100, while iEFO achieves significantly better results than WSA on benchmarks F1, F3–F6, F8, F9, F11, and F12. iEFO outperforms EFO, EMA, CFO, and WSA on 8, 10, 12, and 11 out of 13 test problems. Further, as tabulated in Table 7, for larger problems where *D*=200, the difference between the performance of iEFO and the competitor algorithms is more significant. From Table 7, iEFO is superior to EFO, GSA, and CFO on 11, 10, 13, 12, and 13 out of 13 problems, while iEFO outperforms EMA and WSA on all test instances. This also indicates the effectiveness of iEFO on large problem instances among well-known physics-inspired metaheuristic algorithms. On the other hand, iEFO yields smaller deviation on the majority of the problem with *D*=50, *D*=100, and *D*=200. This also shows that iEFO surpasses all the competitor algorithms in terms of robustness.

**Figure 4:**
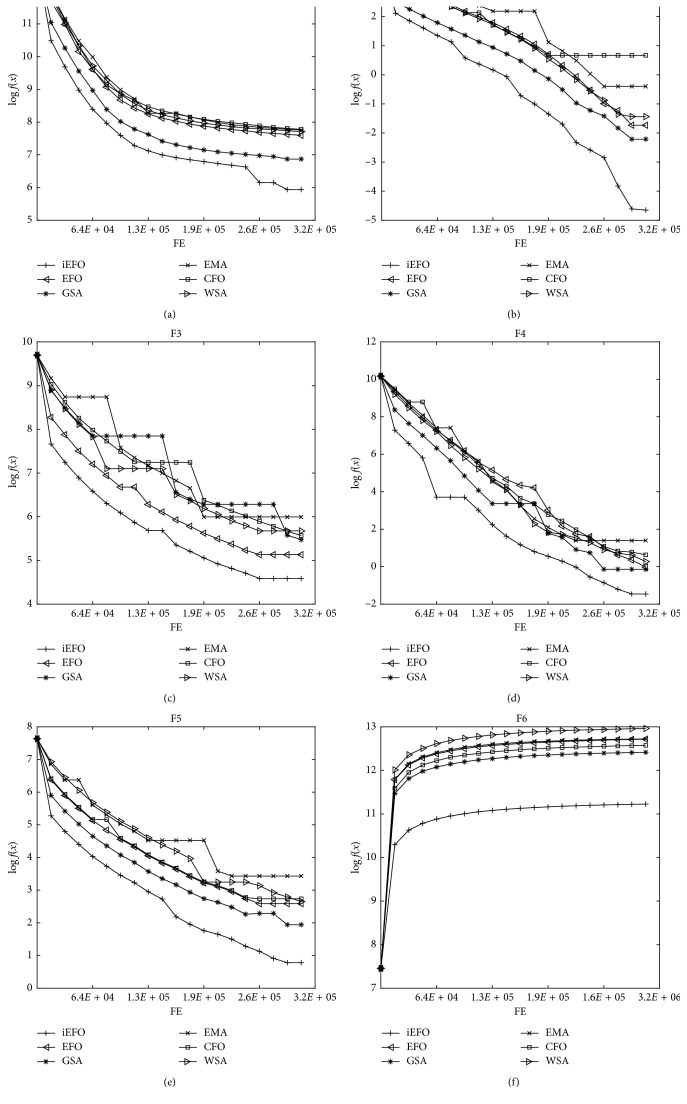
Convergence performance of iEFO and competitor algorithms on *D*=100.

Moreover, the convergence performance of iEFO and competitor algorithms are given in Figure 4 for selected benchmark problems. In Figure 4, the population means are plotted against function evaluation number. It can be concluded from Figure 4 that iEFO indicates better convergence performance than the test algorithms. The results also reveal that the proposed framework appears to be highly competitive in terms of convergence speed.

Lastly, the CPU time results of all algorithms are tabulated in Table 8. Table 8 summarizes the average of CPU times on different dimensions in terms of mean and standard deviation (StdDev). As can be seen from Table 8, the CPU times of iEFO, EFO, GSA, EMA, CFO, and WSA are very close to each other. In other words, there is no significance difference between iEFO and other test algorithms.

Furthermore, the average rankings based on nonparametric Friedman's test of all compared algorithms are given in Table 9. As shown in Table 9, iEFO ranks the first on all dimensions. The test statistics and the *p*-values also indicate the significant difference between iEFO and other algorithms. Overall, the experimental results in this subsection indicate the outperforming performance of iEFO against other physics-inspired algorithms.

### 4.3. Comparison with ABC, PSO, and DE

In this subsection, the performance comparisons on the solution quality are conducted among iEFO and ABC, PSO, and DE. The results shown in Tables 10 [Table tab11]–12 in terms of mean and StdDev of the solutions obtained from 30 independent runs. Similar to the previous subsection, *t*-tests are carried out to test the significance of the results. As described in Table 10, for the benchmark functions with *D*=50, it is worth noting that iEFO is significantly superior to all competitor algorithms on most of the cases. iEFO and ABC show similar performance on F1, F6, F7, F10, and F11, and iEFO performs significantly better than ABC on all other benchmarks. Further, iEFO outperforms PSO and DE on 8 and 6 out of 13 problems, respectively. For the functions with *D*=100, according to the overall performance given in the last row of Table 11, it is clear that iEFO wins in 9, 10, and 10 problems against ABC, PSO, and DE, respectively. iEFO and ABC tie on four functions (F1, F7, F9, and F13), iEFO and PSO tie on three functions (F5, F7, and F10), and similarly there is no significance difference between iEFO and DE on F1, F7, and F10. As described in Table 12, where *D*=200, iEFO superior to ABC, PSO, and DE on 11, 12, and 12 out of 13 functions, respectively. Further, iEFO generally reaches smaller deviations when compared to the test algorithms, which validates the robustness of the proposed iEFO. To intuitively verify the significance difference between iEFO and other three state-of-the-art algorithms, Friedman's tests are carried out similar to the previous subsection.  Table 13 shows Friedman's test rankings test of all compared algorithms. From Table 13, it is clear that iEFO is the best algorithm among ABC, PSO, and DE. Further, the performance of iEFO is even better as the problem size increases.

Summarizing the above statements, iEFO achieves high-quality results in terms of solution quality and robustness when compared to state-of-the-art algorithms, i.e., ABC, PSO, and DE. In other words, the exploitation and exploration of iEFO are well balanced.

### 4.4. Comparison with PSO and DE Variants

This subsection covers the computational results of iEFO against state-of-the-art PSO and DE variants. Since the reported results are taken directly from the corresponding papers, statistical significance tests between algorithms are not carried out. Tables 14 and 15 present computational results. The results for the test cases with *D*=30 are listed in Tables 13 and 14. Some of the test problems are not covered in the comparisons, as corresponding results were not given in the original studies. In Tables 14 and 15, the best mean values are indicated in bold. As can be seen from Table 14, iEFO outperforms FIPS, CLPSO, and HPSO-HVAC on F1–F3, F6, and F13. Further, FIPS is better than iEFO on F4 and HPSO-HVAC is superior to iEFO on F12. All algorithms reach the global optimum for all runs on F11. From Table 15, it is clear that iEFO outperforms JDE and SaDE on all benchmark problems. On the other hand, JADE achieves the best results on F2, F11, and F12. These results also reveal the effectiveness of iEFO when compared to novel PSO and DE variants.

## 5. Conclusion

In this paper, an improved version of the EFO (iEFO) is introduced to solve the global optimization problem. The iEFO enriches the solution search strategy of the original EFO by improving two main steps of the algorithm. First, roulette wheel selection strategy embedded new solution generation function is used to provide more chance to use of fittest electromagnetic particles in the candidate solution generation step. Second, an adaptive control mechanism for the algorithmic parameters is used to increase the selection probability of better solution and reduce the selection probability of worse solution throughout the search process. Besides both two improvements, the boundary control and randomization procedure is modified. To test the performance of the proposed algorithm, a well-known benchmark problem set is used in computational studies. To show the efficiency of the iEFO, three sets of comparisons are carried out. First, the iEFO is compared with original EFO and existing physics-inspired metaheuristic algorithms. Second, three well-known metaheuristic algorithms (ABC, DE, and PSO), whose performances are demonstrated on global optimization problem in many researches, are taken into account. Finally, the iEFO is compared with the improved variants of the DE and PSO. As a result of the extensive statistical analyses, it can be expressed that the proposed iEFO provides efficient solutions and superior to competitor algorithms on most of the problem instances. As a future work, the binary or integer version of the iEFO can be studied for combinatorial problems, such as scheduling problems, vehicle routing problems, knapsack problems, and set covering problems. Further, hybridizing the iEFO with other metaheuristic algorithms will be vital research direction.

## Figures and Tables

**Table 1:** List of the physics-inspired algorithms.

Algorithm	Reference	Year	Inspiration	Reviewed in	
				[9]	[10]
Atom search algorithm	Zhao et al. [6]	2018	Basic molecular dynamics		
Flow regime algorithm	Tahani and Babayan [10]	2018	Concepts of fluid mechanics		
Hydrological cycle algorithm	Wedyan et al. [11]	2017	The continuous movement of water in nature		
Thermal exchange optimization	Kaveh and Dadras [12]	2017	Newton's law of cooling		
Weighted superposition algorithm	Baykasoğlu and Akpinar [13]	2017	Superposition principle and attraction		
Electromagnetic field optimization	Abedinpourshotorban et al. [14].	2016	Attraction-repulsion forces among electromagnets		
Rain water algorithm	Biyanto et al. [15]	2016	The pattern of physically rain water movements		
Ion motion algorithm	Javidy et al. [16]	2015	Ions motion in nature	✓	✓
Colliding bodies optimization	Kaveh and Mahdavi [17]	2014	Collision between objects		
Black hole optimization	Hatamlou [18]	2013	Black hole phenomenon		✓
Dłotko–Specogna	Dłotko and Specogna [19]	2013	Low-frequency electrodynamics		
Ray optimization	Kaveh and Khayatazad [20]	2012	The transition of ray from one medium to another from physics	✓	✓
Water cycle algorithm	Eskandar et al. [21]	2012	Water cycle process and how rivers and streams flow to the sea in the real-world	✓	✓
Galaxy based search algorithm	Shah-Hosseini [22]	2011	The arm of spiral galaxies in the outer space	✓	✓
Gravitational interactions optimization	Flores et al. [23]	2011	Gravitational forces produced by the interaction of the masses of a set of bodies	✓	
Spiral optimization algorithm	Tamura and Yasuda [24]	2011	The analogy of spiral phenomena in nature	✓	
Water flow algorithm	HIEU [25]	2011	Characteristics of water droplets and their erosion capability to overcome obstacles	✓	✓
Charged system search	Kaveh and Talatahari [26]	2010	Coulomb law from electrostatics and the Newtonian laws of mechanics	✓	✓
Gravitation field algorithm	Zheng et al. [27]	2010	Astronomy theory solar nebular disk model of planetary formation	✓	
Artificial physics algorithm	Xie et al. [28]	2009	Newton's second law	✓	✓
Gravitational search algorithm	Rashedi et al. [29]	2009	Law of gravity and mass interactions	✓	✓
Intelligent water drops algorithm	Shah-Hosseini [30]	2009	Natural water drops that flow in rivers	✓	✓
Light ray optimization algorithm	Shen and Li [31]	2009	Optical refraction and reflection of light rays	✓	
Big Crunch optimization	Kripka and Kripka [32]	2008	Cosmological theory is known as closed universe	✓	✓
Magnetic optimization algorithm	Tayarani-N and Akbarzadeh-T [33]	2008	Principles of magnetic field theory	✓	✓
Central force optimization algorithm	[34]	2007	The metaphor of gravitational kinematics	✓	✓
Integrated radiation algorithm	Chuang and Jiang [35]	2007	Gravitational radiation in the curvature of space-time	✓	✓
River formation dynamics algorithm	Rabanal et al. [36]	2007	River formation by water	✓	✓
Big Bang-Big Crunch algorithm	Erol and Eksin [37]	2006	Energy dissipation (Big Bang) and center of mass (Big Crunch)	✓	✓
Particle collision algorithm	Sacco and De Oliveira [38]	2005	Nuclear collision reactions, particularly scattering and absorption	✓	✓
Space gravitational algorithm	Hsiao et al. [39]	2005	Einstein's general theory of relativity	✓	✓
Electromagnetism-like algorithm	Birbil and Fang [40]	2003	Attraction-repulsion mechanism of the sample points	✓	✓
Hysteretic optimization	Zarand et al. [41]	2002	Demagnetization procedure	✓	✓
Harmony search	Geem et al. [42]	2001	Improvisation of the music player		✓
Simulated annealing	Kirkpatrick et al. [43]	1983	Annealing process of molten metals		✓

**Table 2:** Notations used in the EFO.

*N*_emp	Number of EMPs in population
*N*_var	Number of electromagnets of EMP
EMP_*j*_^*i*^	Value of electromagnet *j* in particle *i*, *i*=1,…, *N*_emp, *j*=1,…, *N*_var
EMP_*j*_^New^	Value of electromagnet *j* in new solution, *j*=1,…, *N*_var
*U*_*j*_	Upper bound of the *j* ^th^ electromagnet, *j*=1,…, *N*_var
*L*_*j*_	Lower bound of the *j* ^th^ electromagnet, *j*=1,…, *N*_var
*P*_*j*_	Random index from positive field generated for each electromagnet for the candidate solution, *j*=1,…, *N*_var
*N*_*j*_	Random index from negative field generated for each electromagnet for the candidate solution, *j*=1,…, *N*_var
*K*_*j*_	Random index from neutral field generated for each electromagnet for the candidate solution, *j*=1,…, *N*_var
*P*_field	Portion of population, which belongs to positive field
*N*_field	Portion of population, which belongs to negative field
*P*s_rate	Probability of selecting variables of generated particle
*R*_rate	Probability of changing one variable of a generated particle
*φ*	Golden ratio
rand	Uniform random number rand ∈ (0,1)
rand_int	Integer random number
RI	Index of one electromagnet of generated particle used for randomization step

**Table 3:** Benchmark functions used for the comparisons.

Label	Name	Formulation	Type	Range	*f*(*x* ^*∗*^)
F1	Rosenbrock	$f_{1}\left(\vec{X}\right)=\sum_{i=1}^{D-1}\left[100{\left(x_{i+1}-x_{i}^{2}\right)}^{2}+{\left(x_{i}-1\right)}^{2}\right]$	UN	[−2.048, 2.048]^*D*^	0
F2	Ackley	$f_{2}\left(\vec{X}\right)=-20\;\;\exp\left(-0.2\sqrt{\left(1/D\right)\sum_{i=1}^{D}x_{i}^{2}}\right)-\exp\left(\left(1/D\right)\sum_{i=1}^{D}\cos\left(2\pi x_{i}\right)\right)+20+e$	MS	[−32.768, 32.768]^*D*^	0
F3	Rastrigin	$f_{3}\left(\vec{X}\right)=\sum_{i=1}^{D}\left[x_{i}^{2}-10\cos\left(2\pi x_{i}\right)+10\right]$	MS	[−5.12, 5.12]^*D*^	0
F4	Griewank	$f_{4}\left(\vec{X}\right)=\left(1/4000\right)\sum_{i=1}^{D}x_{i}^{2}-\prod_{i=1}^{D}\cos\left(x_{i}/\sqrt{i}\right)+1$	MN	[−600,600]^*D*^	0
F5	Weierstrass	$f_{5}\left(\vec{X}\right)=\sum_{i=1}^{D}\left(\sum_{k=0}^{k_{\max}}\left[a^{k}\cos\left(2\pi b^{k}\left(x_{i}+0.5\right)\right)\right]\right)-D\sum_{k=0}^{k_{\max}}\left[a^{k}\cos\left(2\pi b^{k}0.5\right)\right],\;\;a=0.5,\;\;b=3,\;\;k_{\max}=20$	MS	[−0.5, 0.5]^*D*^	0
F6	Schwefel 2.26	$f_{6}\left(\vec{X}\right)=\sum_{i=1}^{D}-x_{i}\sin\left(\sqrt{\left|x_{i}\right|}\right)$	MS	[−500,500]^*D*^	−418.98 × *D*
F7	Shifted Sphere	$f_{7}\left(\vec{X}\right)=\sum_{i=1}^{D}z_{i}^{2}-f_{\text{bias}},\;\;z=x-o,\;\;f_{\text{bias}}=-450$	US	[−100,100]^*D*^	*f*_bias_
F8	Shifted Schwefel 1.2	$f_{8}\left(\vec{X}\right)=\sum_{i=1}^{D}\left(\sum_{j=1}^{i}z_{j}\right)+f_{\text{bias}},\;\;z=x-o,\;\;f_{\text{bias}}=-450$	UN	[−100,100]^*D*^	*f*_bias_
F9	Shifted Rosenbrock	$f_{9}\left(\vec{X}\right)=\sum_{i=1}^{D-1}\left(100{\left(z_{i}^{2}-z_{i+1}\right)}^{2}+{\left(z_{i}-1\right)}^{2}\right)+f_{\text{bias}},\;\;z=x-o+1,\;\;f_{\text{bias}}=390$	MN	[−100,100]^*D*^	*f*_bias_
F10	Shifted Rastrigin	$f_{10}\left(\vec{X}\right)=\sum_{i=1}^{D}\left[z_{i}^{2}-10\;\;\cos\left(2\pi z_{i}\right)+10\right]+f_{\text{bias}},z=x-o,\;\;f_{\text{bias}}=-330$	MS	[−5,5]^*D*^	*f*_bias_
F11	Step	$f_{11}\left(\vec{X}\right)=\sum_{i=1}^{D}\left({\left\lfloor x_{i}+0.5\right\rfloor }^{2}\right)$	US	[−100,100]^*D*^	0
F12	Penalized 2	$f_{12}\left(\vec{X}\right)=\left(1/10\right)\left\{\sin^{2}\left(3\pi x_{1}\right)+\sum_{i=1}^{D-1}{\left(x_{i}-1\right)}^{2}\left[1+\;\;\sin^{2}\left(3\pi x_{i+1}\right)\right]+{\left(x_{n}-1\right)}^{2}\left[1+\;\;\sin^{2}\left(2\pi x_{i+1}\right)\right]\right\}+\sum_{i=1}^{D}u\left(x_{i},5,100,4\right)$	MN	[−50,50]^*D*^	0
F13	Alpine	$f_{13}\left(\vec{X}\right)=\overset{D}{\underset{i=1}{\sum }}\left|x_{i}\cdot \sin\left(x_{i}\right)+0.1\cdot x_{i}\right|$	MS	[−10,10]^*D*^	0

**Table 4:** Parameter settings of algorithms.

Algorithm	Parameter setting
EFO [14]	*N*_emp=50, *P*_field=0.1, *N*_field=0.45, *P*s_rate = 0.2, *R*_rate=0.4,
GSA [29]	PopSize=50, *G*_0_=100, *α*=20, *K*_0_=PopSize
EMA [40]	PopSize = 50, *δ*=0.01
CFO [34]	PopSize = 50, *G*=2, *α*=2, *β*=2
WSA [13]	PopSize = 20, *τ*=0.8, sl_*o*_=0.035, *φ*=0.001, *λ*=0.75
ABC [74]	PopSize = 40, Limit =PopSize × *D*
PSO [39]	PopSize = 40, *w*=1.193, *c*1=1.193, *c*2=0.721
DE [75]	PopSize = 40, *F*=0.9, CR=0.9

**Table 5:** Comparisons of iEFO with physics-inspired algorithms on benchmark problems with *D*=50.

Problem	iEFO		EFO			GSA			EMA			CFO			WSA		
	Mean	StdDev	Mean	StdDev	*t*-test	Mean	StdDev	*t*-test	Mean	StdDev	*t*-test	Mean	StdDev	*t*-test	Mean	StdDev	*t*-test
F1	2.64*E* + 01	3.12*E* − 01	5.29*E* + 02	3.20*E* + 02	+	4.90*E* + 01	8.29*E* + 00	+	5.33*E* + 04	2.03*E* + 03	+	8.23*E* + 03	1.23*E* + 02	+	9.23*E* + 03	3.55*E* + 02	+
F2	4.96*E* − 14	8.32*E* − 15	9.07*E* − 13	4.91*E* − 13	+	7.44*E* − 13	3.90*E* − 14	+	2.93*E* − 09	7.19*E* − 12	+	7.33*E* − 12	5.29*E* − 14	+	8.02*E* − 12	4.92*E* − 13	+
F3	4.92*E* − 16	6.93*E* − 16	4.33*E* − 14	6.02*E* − 13	≈	7.03*E* − 14	9.91*E* − 14	+	8.18*E* − 10	4.94*E* − 11	+	7.17*E* − 12	9.62*E* + 12	≈	6.30*E* − 13	5.93*E* − 14	+
F4	1.59*E* − 16	5.63*E* − 17	8.93*E* − 15	2.77*E* − 11	≈	3.29*E* − 15	6.94*E* − 15	+	5.03*E* − 11	4.32*E* − 11	+	3.09*E* − 11	2.59*E* − 14	+	1.92*E* − 11	7.04*E* − 11	≈
F5	6.31*E* − 16	3.08*E* − 17	1.09*E* − 15	8.03*E* − 15	≈	6.93*E* − 15	5.49*E* − 16	+	6.81*E* − 12	8.61*E* − 12	+	7.81*E* − 14	3.99*E* − 16	+	8.32*E* − 16	4.29*E* − 17	+
F6	−2.09*E* + 04	2.41*E* + 00	−1.99*E* + 04	4.30*E* + 01	+	−2.04*E* + 04	3.41*E* + 00	+	−1.99*E* + 04	1.12*E* + 01	+	−2.01*E* + 04	4.31*E* + 02	+	−1.76*E* + 04	2.23*E* + 03	+
F7	−4.50*E* + 02	0.00*E* + 00	−4.50*E* + 02	0.00*E* + 00	≈	−4.50*E* + 02	0.00*E* + 00	≈	−4.50*E* + 02	0.00*E* + 00	≈	−4.50*E* + 02	0.00*E* + 00	≈	−4.50*E* + 02	0.00*E* + 00	≈
F8	2.94*E* + 04	1.59*E* + 03	5.30*E* + 04	4.49*E* + 02	+	3.04*E* + 04	8.02*E* + 03	≈	8.44*E* + 06	5.14*E* + 03	+	6.11*E* + 05	8.92*E* + 03	+	4.92*E* + 05	8.85*E* + 04	+
F9	3.15*E* + 02	2.31*E* + 00	9.51*E* + 03	2.41*E* + 01	+	3.28*E* + 02	1.02*E* + 01	+	3.90*E* + 04	9.54*E* + 02	+	7.32*E* + 04	2.56*E* + 02	+	3.20*E* + 04	4.91*E* + 02	+
F10	−3.30*E* + 02	0.00*E* + 00	−3.30*E* + 02	1.00*E* + 01	≈	−3.30*E* + 02	4.32*E* + 00	≈	−2.88*E* + 02	4.30*E* + 01	+	−3.30*E* + 02	1.20*E* + 01	≈	−3.30*E* + 02	2.36*E* + 02	≈
F11	0.00*E* + 00	0.00*E* + 00	0.00*E* + 00	0.00*E* + 00	≈	0.00*E* + 00	0.00*E* + 00	≈	0.00*E* + 00	0.00*E* + 00	≈	0.00*E* + 00	0.00*E* + 00	≈	0.00*E* + 00	0.00*E* + 00	≈
F12	5.81*E* − 18	8.13*E* − 17	8.84*E* − 17	1.95*E* − 16	+	1.53*E* − 17	6.01*E* − 17	≈	4.33*E* − 13	6.02*E* − 08	≈	5.91*E* − 15	9.27*E* − 19	+	7.20*E* − 17	9.03*E* − 19	+
F13	2.06*E* − 24	1.22*E* − 25	8.52*E* − 23	2.91*E* − 22	≈	3.77*E* − 22	6.58*E* − 23	+	8.58*E* − 15	8.36*E* − 15	+	7.84*E* − 21	3.03*E* − 21	+	4.04*E* − 21	5.39*E* − 22	+
+/−/≈			6/0/7			8/0/5			10/0/3			9/0/4			9/0/4		

**Table 6 tab6:** Comparisons of iEFO with physics-inspired algorithms on benchmark problems with *D*=100.

Problem	iEFO		EFO			GSA			EMA			CFO			WSA		
	Mean	StdDev	Mean	StdDev	*t*-test	Mean	StdDev	*t*-test	Mean	StdDev	*t*-test	Mean	StdDev	*t*-test	Mean	StdDev	*t*-test
F1	9.83*E* + 01	6.39*E* + 00	7.94*E* + 02	5.72*E* + 01	+	1.34*E* + 02	2.11*E* + 01	+	6.88*E* + 05	3.86*E* + 02	+	1.14*E* + 04	6.94*E* + 02	+	2.24*E* + 05	5.06*E* + 02	+
F2	4.03*E* − 11	1.28*E* − 12	8.11*E* − 12	9.48*E* − 11	≈	6.23*E* − 12	4.18*E* − 14	−	8.21*E* − 08	8.31*E* − 09	+	8.21*E* − 11	2.60*E* − 11	+	6.73*E* − 11	4.77*E* − 13	+
F3	7.31*E* − 14	8.23*E* − 15	9.34*E* − 11	9.34*E* − 12	+	6.26*E* − 13	2.10*E* − 13	+	5.54*E* − 09	9.18*E* − 10	+	8.51*E* − 11	4.99*E* − 12	+	3.53*E* − 11	2.28*E* − 12	+
F4	6.04*E* − 14	4.91*E* − 15	4.13*E* − 11	6.49*E* − 13	+	2.95*E* − 13	7.98*E* − 14	+	7.59*E* − 10	3.13*E* − 10	+	2.11*E* − 11	6.82*E* − 12	+	4.18*E* − 11	1.90*E* − 10	≈
F5	2.04*E* − 13	7.17*E* − 13	3.90*E* − 13	1.44*E* − 11	≈	1.01*E* − 12	6.80*E* − 13	+	4.63*E* − 10	9.40*E* − 10	≈	7.43*E* − 12	4.47*E* − 13	+	9.75*E* − 13	4.17*E* − 16	+
F6	−1.12*E* + 04	3.55*E* + 00	−8.40*E* + 03	3.02*E* + 01	+	−9.34*E* + 03	7.35*E* + 00	+	−5.68*E* + 01	6.00*E* + 01	+	−6.31*E* + 03	3.85*E* + 00	+	−8.21*E* + 02	2.07*E* + 01	+
F7	−4.50*E* + 02	8.34*E* − 02	−3.91*E* + 02	4.23*E* + 00	+	−4.50*E* + 02	2.30*E* + 01	≈	−6.20*E* + 01	7.60*E* + 00	+	−6.74*E* + 01	5.46*E* + 00	+	−5.93*E* + 01	5.43*E* + 00	+
F8	8.92*E* + 04	8.34*E* + 04	9.34*E* + 04	7.33*E* + 05	≈	4.73*E* + 05	1.86*E* + 01	+	9.95*E* + 04	5.10*E* + 01	≈	9.98*E* + 06	8.35*E* + 02	+	5.36*E* + 07	5.21*E* + 01	+
F9	9.44*E* + 02	6.93*E* + 01	2.90*E* + 04	8.93*E* + 02	+	3.11*E* + 03	9.36*E* + 01	+	4.09*E* + 05	1.64*E* + 03	+	3.50*E* + 06	8.56*E* + 01	+	3.82*E* + 06	4.24*E* + 02	+
F10	−3.30*E* + 02	5.92*E* − 06	−3.30*E* + 02	5.29*E* − 01	≈	−3.30*E* + 02	4.14*E* − 03	≈	−1.66*E* + 01	3.92*E* − 02	+	−9.01*E* + 01	6.91*E* + 00	+	−2.35*E* + 00	1.59*E* + 00	+
F11	1.35*E* + 01	7.30*E* + 01	5.12*E* + 02	3.20*E* + 02	+	7.13*E* + 01	7.49*E* + 00	+	4.02*E* + 02	3.96*E* + 00	+	2.34*E* + 02	3.31*E* + 00	+	9.83*E* + 03	7.29*E* + 00	+
F12	4.18*E* − 13	1.05*E* − 13	9.12*E* − 12	5.02*E* − 12	+	5.25*E* − 13	4.02*E* − 14	+	8.41*E* − 11	8.40*E* − 12	+	1.15*E* − 12	5.09*E* − 13	+	3.12*E* − 12	7.37*E* − 12	≈
F13	7.07*E* − 20	1.66*E* − 22	7.18*E* − 20	5.27*E* − 15	≈	7.93*E* − 20	9.02*E* − 19	≈	2.12*E* − 15	6.84*E* − 14	≈	3.74*E* − 16	6.60*E* − 15	≈	2.95*E* − 16	1.51*E* − 17	+
+/−/≈			8/0/5			9/1/3			10/0/3			12/0/1			11/0/2		

**Table 7:** Comparisons of iEFO with physics-inspired algorithms on benchmark problems with *D*=200.

Problem	iEFO		EFO			GSA			EMA			CFO			WSA		
	Mean	StdDev	Mean	StdDev	*t*-test	Mean	StdDev	*t*-test	Mean	StdDev	*t*-test	Mean	StdDev	*t*-test	Mean	StdDev	*t*-test
F1	1.17*E* + 02	6.26*E* + 01	3.82*E* + 03	6.86*E* + 01	+	8.95*E* + 04	5.30*E* + 03	+	4.53*E* + 07	3.02*E* + 02	+	4.63*E* + 05	3.96*E* + 02	+	1.41*E* + 07	4.04*E* + 02	+
F2	3.91*E* − 05	8.22*E* − 07	6.50*E* − 04	8.62*E* − 06	+	3.83*E* − 05	3.06*E* − 05	≈	6.01*E* − 04	9.02*E* − 06	+	2.68*E* − 04	2.28*E* − 04	+	4.96*E* − 05	3.81*E* − 07	+
F3	1.32*E* − 04	3.91*E* − 06	5.96*E* − 03	7.74*E* − 07	+	8.89*E* − 03	1.37*E* − 04	+	1.24*E* − 03	9.60*E* − 04	+	2.29*E* − 01	4.66*E* − 03	+	8.69*E* − 02	7.94*E* − 05	+
F4	9.81*E* − 10	4.81*E* − 09	6.80*E* − 09	4.50*E* − 08	≈	3.57*E* − 09	6.54*E* − 10	+	3.04*E* − 08	3.92*E* − 10	+	2.05*E* − 08	3.85*E* − 07	≈	2.82*E* − 09	6.58*E* − 11	+
F5	1.85*E* − 04	7.49*E* − 05	5.86*E* − 03	3.53*E* − 04	+	6.26*E* − 03	3.11*E* − 04	+	4.21*E* − 03	9.94*E* − 04	+	5.59*E* − 03	7.17*E* − 04	+	4.96*E* − 03	3.74*E* − 05	+
F6	−8.12*E* + 04	3.14*E* + 01	−5.92*E* + 03	5.57*E* + 02	+	−7.94*E* + 02	2.84*E* + 01	+	−3.86*E* + 03	8.43*E* + 01	+	−6.27*E* + 03	6.49*E* + 01	+	−2.33*E* + 04	4.53*E* + 00	+
F7	3.12*E* + 05	4.73*E* + 02	1.92*E* + 06	5.60*E* + 03	+	9.37*E* + 05	4.36*E* + 02	+	5.02*E* + 08	6.75*E* + 02	+	5.07*E* + 05	7.49*E* + 02	+	2.87*E* + 06	7.99*E* + 02	+
F8	3.44*E* + 05	7.22*E* + 03	9.15*E* + 07	6.08*E* + 03	+	2.80*E* + 06	9.36*E* + 03	+	7.06*E* + 06	1.25*E* + 03	+	8.33*E* + 08	3.84*E* + 02	+	1.48*E* + 09	5.68*E* + 04	+
F9	4.38*E* + 06	8.04*E* + 04	9.92*E* + 06	7.80*E* + 02	+	9.48*E* + 06	9.20*E* + 02	+	6.28*E* + 06	9.62*E* + 03	+	1.43*E* + 08	7.55*E* + 03	+	1.33*E* + 08	4.31*E* + 03	+
F10	4.33*E* + 02	3.34*E* + 01	5.24*E* + 02	1.24*E* + 01	+	7.70*E* + 02	8.65*E* + 02	≈	7.16*E* + 02	7.21*E* + 00	+	6.81*E* + 02	5.84*E* + 01	+	1.80*E* + 03	7.30*E* + 01	+
F11	3.28*E* + 02	8.30*E* + 01	4.64*E* + 03	8.51*E* + 01	+	7.10*E* + 02	6.19*E* + 01	+	2.54*E* + 04	5.28*E* + 03	+	1.56*E* + 04	4.93*E* + 02	+	6.95*E* + 04	1.35*E* + 02	+
F12	1.82*E* − 11	7.85*E* − 11	1.05*E* − 09	9.20*E* − 10	+	1.50*E* − 08	8.48*E* − 10	+	2.07*E* − 09	4.29*E* − 11	+	3.26*E* − 08	7.44*E* − 13	+	1.02*E* − 10	4.43*E* − 11	+
F13	2.03*E* − 18	5.73*E* − 15	7.37*E* − 16	1.66*E* − 14	≈	2.32*E* − 17	5.56*E* − 14	≈	1.37*E* − 13	9.61*E* − 14	+	8.03*E* − 15	6.90*E* − 15	+	2.16*E* − 14	8.92*E* − 16	+
+/−/≈			11/0/2			10/0/3			13/0/0			12/0/1			13/0/0		

**Table 8:** CPU time comparison of iEFO and competitor algorithms.

Algorithm	*D* = 50		*D* = 100		*D* = 200	
	Mean	StdDev	Mean	StdDev	Mean	StdDev
iEFO	13.83	1.43	16.73	1.14	18.54	1.92
EFO	13.11	2.08	15.84	1.56	17.21	1.77
GSA	12.91	1.74	15.18	1.82	18.37	1.98
EMA	13.50	1.68	15.29	1.70	17.84	2.04
CFO	14.79	1.97	16.75	1.94	18.92	1.96
WSA	12.46	1.56	15.22	1.81	17.33	1.89

**Table 9:** Friedman-test results for the iEFO and physics-inspired algorithms.

Rank	Average			Dimension (*D*=50)		Dimension (*D*=100)		Dimension (*D*=200)	
	Algorithm	Ranking		Algorithm	Ranking	Algorithm	Ranking	Algorithm	Ranking
1	iEFO	1.29		iEFO	1.54	iEFO	1.27	iEFO	1.08
2	GSA	2.78		GSA	2.62	GSA	2.27	GSA	3.46
3	EFO	3.29		EFO	3.12	EFO	3.15	EFO	3.62
4	CFO	4.28		WSA	3.92	CFO	4.15	WSA	4.23
5	WSA	4.33		CFO	4.38	WSA	4.85	EMA	4.31
6	EMA	5.01		EMA	5.42	EMA	5.31	CFO	4.31
			Statistic	35.05		45.01		35.05	
			*p* value	<0.001		<0.001		<0.001	

**Table 10:** Comparisons of iEFO with ABC, PSO, and DE on benchmark problems with *D*=50.

Problem	iEFO		ABC			PSO			DE		
	Mean	StdDev	Mean	StdDev	*t*-test	Mean	StdDev	*t*-test	Mean	StdDev	*t*-test
F1	2.64*E* + 01	3.12*E* − 01	3.02*E* + 01	1.11*E* + 01	≈	3.54*E* + 01	1.44*E* + 01	+	3.32*E* + 01	1.05*E* + 01	+
F2	4.96*E* − 14	8.32*E* − 15	1.21*E* − 13	1.51*E* − 14	+	7.90*E* − 14	1.89*E* − 14	+	8.06*E* − 14	8.95*E* − 15	+
F3	4.92*E* − 16	6.93*E* − 16	2.31*E* − 11	7.29*E* − 12	+	8.44*E* − 14	6.86*E* − 12	≈	9.11*E* − 14	1.95*E* − 12	≈
F4	1.59*E* − 16	5.63*E* − 17	4.88*E* − 12	2.85*E* − 13	+	1.04*E* − 13	2.11*E* − 13	+	5.89*E* − 16	1.55*E* − 13	≈
F5	6.31*E* − 16	3.08*E* − 17	3.66*E* − 14	2.01*E* − 14	+	7.70*E* − 15	1.23*E* − 16	+	8.37*E* − 15	8.59*E* − 15	+
F6	−2.09*E* + 04	2.41*E* + 00	−2.09*E* + 04	6.01*E* + 00	≈	−2.09*E* + 04	6.01*E* + 00	≈	−2.09*E* + 04	4.15*E* + 00	≈
F7	−4.50*E* + 02	0.00*E* + 00	−4.50*E* + 02	6.90*E* − 14	≈	−4.50*E* + 02	7.11*E* − 14	≈	−4.50*E* + 02	8.27*E* − 15	≈
F8	2.94*E* + 04	1.59*E* + 03	3.29*E* + 04	1.43*E* + 03	+	3.10*E* + 04	1.11*E* + 03	+	3.89*E* + 04	1.44*E* + 03	+
F9	3.15*E* + 02	2.31*E* + 00	5.88*E* + 02	4.52*E* + 00	+	4.19*E* + 02	5.01*E* + 00	+	3.87*E* + 02	2.90*E* + 00	+
F10	−3.30*E* + 02	0.00*E* + 00	−3.30*E* + 02	3.02*E* − 14	≈	−3.30*E* + 02	2.86*E* − 14	≈	−3.30*E* + 02	7.40*E* − 15	≈
F11	0.00*E* + 00	0.00*E* + 00	0.00*E* + 00	0.00*E* + 00	≈	0.00*E* + 00	0.00*E* + 00	≈	0.00*E* + 00	0.00*E* + 00	≈
F12	5.81*E* − 18	8.13*E* − 17	5.44*E* − 17	9.44*E* − 17	+	9.05*E* − 17	5.68*E* − 17	+	1.59*E* − 17	6.27*E* − 16	≈
F13	2.06*E* − 24	1.22*E* − 25	3.25*E* − 23	2.65*E* − 23	+	6.13*E* − 24	8.66*E* − 24	+	7.95*E* − 24	9.06*E* − 24	+
+/−/≈			8/0/5			8/0/5			6/0/7		

**Table 11 tab11:** Comparisons of iEFO with ABC, PSO, and DE on benchmark problems with *D*=100.

Problem	iEFO		ABC			PSO			DE		
	Mean	StdDev	Mean	StdDev	*t*-test	Mean	StdDev	*t*-test	Mean	StdDev	*t*-test
F1	9.83*E* + 01	6.39*E* + 00	1.44*E* + 02	4.71*E* + 02	≈	1.15*E* + 03	9.00*E* + 02	+	1.10*E* + 02	2.31*E* + 03	≈
F2	4.03*E* − 12	1.28*E* − 12	6.34*E* − 07	7.44*E* − 08	+	3.04*E* − 09	8.43*E* − 11	+	6.41*E* − 11	4.02*E* − 12	+
F3	7.31*E* − 14	8.23*E* − 15	2.43*E* − 05	1.00*E* − 06	+	5.84*E* − 09	3.61*E* − 10	+	1.21*E* − 12	7.26*E* − 13	+
F4	6.04*E* − 14	4.91*E* − 15	1.11*E* − 04	7.33*E* − 05	+	4.22*E* − 09	2.68*E* − 10	+	3.54*E* − 12	7.22*E* − 14	+
F5	2.04*E* − 13	7.17*E* − 13	1.92*E* − 04	8.90*E* − 15	+	3.96*E* − 13	4.92*E* − 14	≈	5.40*E* − 11	3.44*E* − 14	+
F6	−1.12*E* + 04	3.55*E* + 00	−4.02*E* + 03	2.11*E* + 02	+	−4.11*E* + 03	1.53*E* + 02	+	−4.19*E* + 03	6.24*E* + 01	+
F7	−4.50*E* + 02	8.34*E* − 02	−4.50*E* + 02	5.32*E* + 01	≈	−4.50*E* + 02	8.53*E* + 00	≈	−4.50*E* + 02	4.90*E* + 00	≈
F8	8.92*E* + 04	8.34*E* + 04	2.35*E* + 05	3.05*E* + 04	+	1.65*E* + 05	1.05*E* + 04	+	1.66*E* + 05	7.77*E* + 03	+
F9	9.44*E* + 02	6.93*E* + 01	9.01*E* + 02	2.11*E* + 02	≈	4.01*E* + 03	2.98*E* + 01	+	4.52*E* + 03	9.01*E* + 01	+
F10	−3.30*E* + 02	5.92*E* − 06	−3.22*E* + 02	3.68*E* − 05	+	−3.30*E* + 02	7.20*E* − 07	≈	−3.30*E* + 02	6.82*E* − 14	≈
F11	1.35*E* + 01	7.30*E* + 01	5.37*E* + 01	8.73*E* + 00	+	6.51*E* + 01	6.89*E* + 00	+	5.43*E* + 01	9.52*E* + 00	+
F12	4.18*E* − 13	1.05*E* − 13	6.84*E* − 11	5.32*E* − 13	+	7.16*E* − 13	3.18*E* − 14	+	1.15*E* − 12	3.91*E* − 13	+
F13	7.07*E* − 21	1.66*E* − 22	9.05*E* − 19	3.04*E* − 18	≈	9.04*E* − 20	1.22*E* − 21	+	7.89*E* − 19	1.06*E* − 19	+
+/−/≈			9/0/4			10/0/3			10/0/3		

**Table 12:** Comparisons of iEFO with ABC, PSO, and DE on benchmark problems with *D*=200.

Problem	iEFO		ABC			PSO			DE		
	Mean	StdDev	Mean	StdDev	*t*-test	Mean	StdDev	*t*-test	Mean	StdDev	*t*-test
F1	1.17*E* + 02	6.26*E* + 01	4.32*E* + 02	1.04*E* + 02	+	4.47*E* + 02	6.20*E* + 01	+	4.58*E* + 02	3.94*E* + 01	+
F2	3.91*E* − 05	8.22*E* − 07	4.44*E* − 02	5.28*E* − 03	+	6.68*E* − 05	8.54*E* − 06	+	1.47*E* − 04	6.23*E* − 05	+
F3	1.32*E* − 04	3.91*E* − 03	3.17*E* + 01	6.03*E* + 00	+	6.51*E* + 00	2.44*E* + 00	+	3.91*E* − 01	6.16*E* − 02	+
F4	9.81*E* − 10	4.81*E* − 09	5.25*E* − 04	8.30*E* − 06	+	1.79*E* − 08	7.19*E* − 09	+	6.05*E* − 08	3.65*E* − 09	+
F5	1.85*E* − 04	7.49*E* − 05	1.51*E* − 01	3.55*E* − 03	+	9.76*E* − 03	1.13*E* − 04	+	1.83*E* − 02	7.04*E* − 03	+
F6	−8.12*E* + 04	3.14*E* + 01	−7.59*E* + 04	5.01*E* + 02	+	−8.15*E* + 04	4.99*E* + 03	≈	−7.37*E* + 04	8.34*E* + 02	+
F7	6.12*E* + 05	4.73*E* + 02	8.10*E* + 05	1.46*E* + 04	+	8.73*E* + 05	9.49*E* + 04	+	8.62*E* + 05	2.09*E* + 04	+
F8	3.44*E* + 05	7.22*E* + 03	2.55*E* + 06	5.29*E* + 04	+	2.93*E* + 06	5.18*E* + 05	+	2.98*E* + 06	7.35*E* + 05	+
F9	4.38*E* + 10	8.04*E* + 04	8.07*E* + 11	5.04*E* + 07	+	7.87*E* + 11	4.91*E* + 06	+	7.61*E* + 11	4.33*E* + 05	+
F10	4.33*E* + 02	3.34*E* + 01	1.57*E* + 02	2.49*E* + 03	≈	3.76*E* + 03	7.37*E* + 02	+	3.76*E* + 03	2.54*E* + 02	+
F11	3.28*E* + 02	8.30*E* + 01	8.15*E* + 04	9.90*E* + 02	+	5.39*E* + 03	3.04*E* + 02	+	1.04*E* + 03	9.46*E* + 02	+
F12	1.82*E* − 11	7.85*E* − 10	2.01*E* − 09	5.33*E* − 12	+	4.88*E* − 11	9.92*E* − 12	≈	6.22*E* − 11	9.04*E* − 13	≈
F13	2.03*E* − 18	5.73*E* − 18	5.82*E* − 16	4.40*E* − 15	≈	4.72*E* − 16	2.81*E* − 17	+	1.92*E* − 17	4.23*E* − 18	+
+/−/≈			11/0/2			11/0/2			12/0/1		

**Table 13:** Friedman-test results for the iEFO and ABC, PSO, and DE.

Rank	Average			Dimension (*D*=50)		Dimension (*D*=100)		Dimension (*D*=200)	
	Algorithm	Ranking		Algorithm	Ranking	Algorithm	Ranking	Algorithm	Ranking
1	iEFO	1.29		iEFO	1.46	iEFO	1.27	iEFO	1.15
2	PSO	2.67		PSO	2.62	DE	2.58	PSO	2.65
3	DE	2.74		DE	2.69	PSO	2.73	DE	2.96
4	ABC	3.29		ABC	3.23	ABC	3.42	ABC	3.23
			Statistic	12.97			18.92		20.15
			*p* value	0.009			0.001		<0.001

**Table 14:** Comparisons of iEFO with PSO variants on benchmark problems with *D*=30.

Problem	MaxFE	iEFO		FIPS		HPSO-TVAC		CLPSO	
		Mean	StdDev	Mean	StdDev	Mean	StdDev	Mean	StdDev
F1	200,000	**5.23*E* + 00**	2.34*E* + 00	2.51*E* + 01	5.10*E* − 01	2.39*E* + 01	2.65*E* + 01	1.13*E* + 01	9.85*E* + 00
F2	200,000	**9.19*E* − 15**	5.90*E* − 14	2.33*E* − 07	7.19*E* − 08	7.29*E* − 14	3.00*E* − 14	3.66*E* − 07	7.57*E* − 08
F3	200,000	**7.02*E* − 05**	3.48*E* − 05	6.51*E* + 01	1.33*E* + 01	9.43*E* + 00	3.48*E* + 00	9.05*E* − 05	1.25*E* − 04
F4	200,000	9.55*E* − 12	1.82*E* − 13	**9.01*E* − 12**	1.84*E* − 11	9.75*E* − 03	8.33*E* − 03	9.02*E* − 09	8.57*E* + 09
F6	200,000	**8.02*E* − 05**	7.61*E* − 04	9.93*E* + 02	5.09*E* + 02	1.59*E* + 03	3.26*E* + 02	3.82*E* − 04	1.28*E* − 05
F11	200,000	0.00*E* + 00	0.00*E* + 00	0.00*E* + 00	0.00*E* + 00	0.00*E* + 00	0.00*E* + 00	0.00*E* + 00	0.00*E* + 00
F12	200,000	3.90*E* − 20	4.50*E* − 18	2.70*E* − 14	1.57*E* − 14	**2.79*E* − 28**	2.18*E* − 28	1.25*E* − 12	9.45*E* − 12
F13	200,000	**5.23*E* + 00**	2.34*E* + 00	2.51*E* + 01	5.10*E* − 01	2.39*E* + 01	2.65*E* + 01	1.13*E* + 01	9.85*E* + 00

**Table 15:** Comparisons of iEFO with DE variants on benchmark problems with *D*=30.

Problem	MaxFE	iEFO		JDE		JADE		SaDE	
		Mean	StdDev	Mean	StdDev	Mean	StdDev	Mean	StdDev
F1	300,000	**2.94*E* − 01**	6.23*E* *−* 01	1.30*E* + 01	1.40*E* + 01	3.20*E* *−* 01	1.10*E* + 00	2.10*E* + 01	7.70*E* + 01
F2	50,000	5.12*E* − 09	8.24*E* *−* 08	2.37*E* *−* 04	7.10*E* *−* 05	**3.35*E* − 09**	2.84*E* *−* 09	3.81*E* *−* 06	8.26*E* *−* 07
F3	100,000	**1.63*E* − 09**	5.22*E* *−* 09	2.37*E* *−* 04	7.10*E* *−* 05	3.35*E* *−* 09	2.84*E* *−* 09	3.81*E* *−* 06	8.26*E* *−* 07
F4	50,000	**9.48*E* − 10**	7.19*E* *−* 09	7.29*E* *−* 06	1.05*E* *−* 05	1.57*E* *−* 08	1.09*E* *−* 07	2.52*E* *−* 09	1.24*E* *−* 09
F6	100,000	**7.92*E* − 11**	4.20*E* *−* 11	1.70*E* *−* 10	2.62*E* *−* 10	2.62*E* *−* 04	3.59*E* *−* 04	1.13*E* *−* 08	1.08*E* *−* 08
F11	10,000	9.49*E* + 00	3.11*E* + 00	6.13*E* + 02	1.72*E* + 02	**5.62*E* + 00**	1.87*E* + 00	5.07*E* + 01	1.34*E* + 01
F12	50,000	1.99*E* *−* 10	9.19*E* *−* 11	1.80*E* *−* 05	1.42*E* *−* 05	**1.87*E* − 10**	1.09*E* *−* 09	1.93*E* *−* 09	1.53*E* *−* 09
F13	300,000	**5.38*E* − 10**	3.61*E* *−* 09	6.08*E* *−* 10	8.36*E* *−* 10	2.78*E* *−* 05	8.43*E* *−* 06	2.94*E* *−* 06	3.47*E* *−* 06

## Data Availability

The data used to support the findings of this study are included within the article.
